# Capturing the holistic profile of high performance Olympic weightlifting development

**DOI:** 10.3389/fspor.2022.986134

**Published:** 2022-09-30

**Authors:** Dior N. Jnr Anderson, Victoria Mary Gottwald, Gavin Peter Lawrence

**Affiliations:** ^1^Talent Pathway iD Limited, Gaerwen, United Kingdom; ^2^School of Human and Behavioural Sciences, College of Human Sciences, Bangor University, Bangor, United Kingdom

**Keywords:** talent development, expertise, coaching, performance, machine learning

## Abstract

Recent expertise development studies have used retrospective recall methods to explore developmental biographies and/or practice histories of current or past athletes. This methodological approach limits the generalizability and trustworthiness of findings. As such, a gap exists for research exploring key multidisciplinary features in athlete development using prospective longitudinal research designs. The present research aimed to holistically model the development of talent in Olympic Weightlifting using such a design. We observed the holistic profiles of 29 junior weightlifting athletes longitudinally over a 10-month period, and subsequently classified six of the 23 athletes as high performing based on their performances in competitions up to 12 months following the study. This holistic profile was based on a framework of expertise development themes: (1) demographics and family sport participation, (2) anthropometrics and physiological factors, (3) psychosocial profiling, (4) sport participation history, and (5) weightlifting specific practice activities. A summary model was produced which selected a critical set of nine features that classified group membership with 91% average accuracy. Odds ratio calculations uncovered discriminating features in the holistic profiles of performance groups, from which empirically derived logical statements could inform the description of high-performance attainment.

## Introduction

The development of high performance in sport, stems from a dynamic interplay of a multitude of features (1). However, it is in capturing this interplay, that poses logistical problems for the practitioner and policy maker. Furthermore, problems exist when trying to determine which features appear to be more influential than others, particularly when consolidating past research that has (a) predominantly studied factors influencing performance development in relative degrees of isolation and (b) used statistical approaches that are best suited to experimental and epidemiological research.

In a recently published position stand commissioned by UK Sport, the quality of existing evidence from a broad range of factors influencing the attainment of elite sports performance was explored and recommendations for policy makers and practitioners outlined (2). Whilst proposing avenues for future research, Rees et al. invited research to embrace the complexity and multidisciplinary nature of talent development. This review has since given rise to a recent body of research that has utilized cutting-edge machine learning analytics to approach this problem (3–6). This machine learning approach has allowed for the selection of a critical set of features in the developmental biographies of athletes that best discriminate between two pre-determined athlete groups (e.g., super-elite vs. elite). This critical set can then be used to inform the narrative that best describes the attainment of high performance for the population of interest. When underpinned by a theoretically driven expertise or talent development framework, this approach enables for a much richer mechanism for conceptualizing expertise development. The advent of machine learning algorithms and advanced data handling procedures, now makes it possible for researchers to begin exploring important relationships by deploying algorithms that explore the relative importance of a multitude of attributes simultaneously. The selection of the critical features can then be determined from this analysis. Moreover, the accuracy with which this critical subset best represents a particular problem can also be assessed, which enables for the assessment of a “model's” performance in real world expectations. Additionally, since data science techniques are in the advent of big data, the potential breadth in exploring the dynamic development of expertise is now as wide as it has ever been.

Nevertheless, the current body of research using these techniques has predominantly explored differences in athletes who were selected from a range of different Olympic sports (3, 4), and as such the themes that have emerged from these findings may not be best suited to the specific characteristics of a single sport. To date, only two studies have explored the multidisciplinary determinants of expertise development within a single sport (5, 6); both in the sport of cricket. In 2019, Jones et al. (5) investigated the relative contributions of a set of 93 multidisciplinary attributes on the development of elite performance attainment in cricket spin bowlers and found that a subset of 12 of the 93 attributes classified elite status with 100% accuracy. One year later and using a similar research design, Jones et al. (6) were able to classify super elite batting status with an accuracy of 95% from a subset of only 18 development features from an original set of 693. Interestingly, whilst both the final models retained the multidisciplinary nature of expertise development, both were very different despite being in the same sport. These differences further highlight the need for sport (even discipline) specific approaches when adopting the holistic development methodology. Furthermore, it should be noted that Jones et al. (5, 6), along with Güllich et al. (3) and Hardy et al. (4), adopt retrospective recall methods when exploring these unique developmental biographies and practice and training histories of successful athletes. Whilst fruitful, research has identified limitations of this approach associated with (a) an athlete's past success leading to biased recall perceptions and (b) the contribution of the athletes' physiological and psychosocial profiles during different ages and stages of development is difficult to determine (6, 7). As such, a gap in the current literature exists for research that not only explores the key multidisciplinary features of expertise development but also does so using a prospective longitudinal research design and appropriate statistical methods (i.e., machine learning).

The purpose of the current study was to prospectively explore the features that characterize the development of high performance in youth athletes from a single sport using a prospective and longitudinal design. We observed the development of a group of youth and junior weightlifting athletes over a 2 year period, whilst holistically profiling each athlete from a variety of features. The present study adopted a deductive approach to inform a series of multidisciplinary themes identified as being integral factors underpinning talent development: (a) demographics and family; (b) athlete physiological profiles; (c) athlete psychosocial profiles; (d) sporting history and weightlifting specific involvement; and (e) weightlifting specific practice activities. This was based on Rees et al.'s (2) review of current knowledge on the development of the world's best sporting talent as well as consideration of current understanding of factors influencing talent development [see also (8)].

More specifically, demographics stems from the multitude of research surrounding the sibling effect, birthplace effect, birthdate effect, schooling, and developmental environment on expertise and talent development [e.g., (9–11)]. Anthropometrics from the nature-based literature [e.g., (12)]. Psychosocial factors were selected based upon Hardy et al.'s (4) empirical research that revealed the psychosocial attributes that best discriminated between two predetermined athlete groups. Here, these were super elite (serial medal winning Olympic athletes) and elite (single medal winning Olympic athletes). The psychosocial attributes that best discriminated between these groups formed the basis of the theoretical framework in the current study. These were all underpinned by current understanding of psychological determinants of expertise [for a review see (2)]. The sporting history section of the framework stems from research pertaining to the influence of sampling, specialization, deliberate practice, and deliberate play and stems from a number of talent development frameworks, e.g., deliberate practice (13), long-term athlete development [LTAD; (14)], developmental model of sports participation [DMSP; (15)], differentiated model of giftedness and talent (16), and athletic skills model (17). The detailed practice and training histories aspect of the framework were based around the skill acquisition literature and included age and stage of development experiences associated with practice volumes and proportions of internal and external focus of attention, vicarious experiences, constant and variable practice, blocked and random practice, whole and part practice, consequences and pressure training, prescriptive vs. constraints practice, representative practice and feedback types (prescriptive vs. constraint based) [see (5, 6, 18)]. These themes were chosen on the basis that there is extensive research for each on their influence on skill development and learning, which is clearly an important tenant of talent development. Furthermore, the governing body contributed to methodological development *via* applied expertise and were provided an opportunity to check and challenge measures adopted based on value for the sport. These factors were not by any means considered exhaustive but inclusive of key determinants of expertise and collectively totaled 648 variables.

The aims of the current study were four-fold. Firstly, we adopted a prospective approach to address limitations of retrospective recall; secondly, we employed a longitudinal approach to better account for the dynamic nature of talent development; thirdly, we utilized a multidisciplinary approach alongside sophisticated machine learning techniques to investigate the complex interplay between psychosocial, physical, and skill acquisition related factors that account for long-term athlete development; and finally, we employed a sport specific model to ensure findings were most reflective of the nuances of Olympic weightlifting.

## Methods

### Participants

Twenty-nine youth and junior weightlifting athletes (21 males, mean age 15.3 ± 1.71; 8 females, mean age 15.8 ± 1.98 years) participated in the current study. All athletes were registered to and attended regular training at a weightlifting club that was affiliated to the national governing body for weightlifting in Wales. All athletes were nominated by their weightlifting coach to participate in the study before being formally invited to participate. Ethical approval for the study was granted by the institutional ethics committee. All participants provided consent to participate.

### Measures

A total of 648 variables were collected which explored the following themes: (a) demographics and family information; (b) athlete physiological profile; (c) athlete psychosocial profile; (d) sporting history and weightlifting specific involvement; and (e) weightlifting specific practice activities. These features are listed in Table 1.

**Table 1:** Features used as part of the multidimensional profiling.

**1. Demographics and familial sport participation**
***Familial sport participation:*** Parental involvement in sport and experience in weightlifting, same sex sibling, older same sex sibling, same sex sibling experience in weightlifting
***Homeplace throughout development:*** Population of longest residing homeplace between 6 and 12 years, population density of longest residing homeplace between 6 and 12 years, population of longest residing homeplace between 13 and 15 years, town population of longest residing homeplace between 13 and 15 years, times relocated throughout development
***Schooling:*** Attended sport school between 6 and 12 years, attended sport school between 13 and 15 years, school main place for sport participation between 6 and 12 years, school main place for sport participation between 13 and 15 years
***Relative age:*** Month of birth (1 = January 12 = December), birth quarter [calendar and school; Q1 = Jan-Mar (calendar), Q1 = Sept – Nov (school)], relative age to nearest aged sibling (in days)
**2. Physiology and anthropometrics (variables were controlled for age and gender):**
***Body composition:*** BMI, body fat percentage, fat weight (kg), lean weight (kg), dry lean weight, body water percentage, Total Body Water (L)
***Body segment ratios:*** Upper arm length, forearm length, total arm length, thigh length, tibia length, total leg length, torso length, tibia to height, thigh to height, torso to height, upper arm to height, forearm to height, hand to height, 2D:4D ratio
***Skeletal muscle strength:*** Left hand grip strength, right hand grip strength, hand grip strength asymmetry, back squat to body weight ratio, front squat to body weight ratio
***Stretch shortening cycle utilization:*** Countermovement jump height, squat jump height, peak power [Sayers equation; (50)], peak power [Duncan equation; (51)], standing broad jump distance
***Mobility/trunk stability:*** Body angles during overhead squat test: ankle (relative to horizonal), thigh (relative to horizontal), torso (relative to horizontal), ankle to torso ratio, thigh to ankle ratio, torso to ankle ratio
**3. Psychosocial characteristics (1 to 7-point Likert scale)**
***Achievement motivation:*** mastery approach and avoidance, performance approach, performance avoidance
***Athlete behavior:*** commitment to training, relative importance of sport, total preparation for competition, relative importance sport, passion for weightlifting: harmonious passion, obsessive passion
***Athlete personality:*** conscientiousness, openness to experience, agreeableness, extraversion, emotional stability, ruthlessness and selfishness. Perfectionism: perfectionist strivings, concern over mistakes, perceived parental pressure, perceived coach pressure, doubts about actions, organization
**4. Sport history and weightlifting specific involvement**
***Sport involvement (between 6 and 12 years, 13–15 years):*** Years involved in each of the following sports: athletics, badminton, basketball, boxing, cricket, CrossFit, dance, football, golf, gymnastics, handball, hockey, horse riding, martial arts, motorsports, mountain biking, rounders, rowing, rugby, swimming, tennis, trampoline; years between 6 and 12 years involved in individual sports, team sports, and cgs sports; total number of sports; years between 13 and 15 years involved in individual sports, team sports, and cgs sports; total number of sports
***Weightlifting specific and related involvement (between 6 and 12 years, 13–15 years):*** Number of competitions per year, exposure to competition (h/year), time spent in competition (h/year), flexibility/mobility training (h/week), number of months involved in weightlifting training (h/week), weightlifting specific practice (h/week), strength and conditioning training (h/week)
**5.Microstructure of practice**
***Sport involvement (between 6 and 12 years, 13–15 years):*** Deliberate practice vs. play, mental skills training, vicarious experiences, conveying of information, whole/part practice, constant vs. varied practice, specificity of practice, focus of attention, prescriptive vs. constraints coaching

### Procedure

Testing sessions involved the athletes completing the physical testing battery followed by questionnaires and short, 15–20 min interviews with the lead researcher. At the end of the baseline testing period, athletes were instructed to participate in their regular training program as normal for the next 10 months, after which the second round of testing would commence. Data from the athletes' competitive performances was collected throughout the study and for a further 12 months after the second round of testing. This data was sourced from the Weightlifting Wales (https://www.weightlifting.wales) or British Weightlifting (https://britishweightlifting.org) webpages, or in cases for any international competitions, the International Weightlifting Federation's (IWF; https://www.iwf.net) or European Weightlifting Federation's websites (EWF; https://www.ewfed.com). This data included the recorded snatch, clean and jerk and total weight lifted in each competition, and the rank position for respective weight class. The number of competitions per athlete was also included.

### Data analysis

#### Group classification

British percentile calculations were calculated for each athlete's recorded snatch, clean and jerk, and total at each competition. This was performed to establish each athlete's respective score against a population norm. Performance classifications were then assigned to each athlete based on whether their performance was within the top 80th percentile of British performances for their respective age group and bodyweight classification. This resulted in a total of 23 athletes that were classified as low performance (17 males, 6 females, mean age: 15.1 ± 1.5), and six athletes classified as high performance (4 males, 2 females; mean age: 16.6 ± 1.5) by the second round of testing (T2), respectively. These groups were then used as the classification groups for subsequent machine learning analyses.

#### Machine learning

Machine learning was implemented in the current study to provide a set of rules from which group membership could be best classified. Similar to previous machine learning talent research (3–6), a multi-level methodology was employed. Specifically, we employed parameter optimization, calculation of odds ratios, feature selection, and finally feature classification. The goal of machine learning is to analyze a wide number of features (i.e., our 648 variables) and identify those that best distinguish between two classes of objects (i.e., our high performing and low performing groups of athletes). The next stage is to employ multiple different procedures (or “algorithms”) to classify athletes accordingly. To be conservative, we employed four different conventional algorithms to achieve this: Naïve Bayes, J48 decision tree, Support Vector Machine and K-nearest neighbor. The higher the number of algorithms that agree on the features needed to distinguish between the groups, the higher the confidence we can have in the results. Thus, features that appeared in the top 20 of all four algorithms were classed as high in importance, those that appeared in three of the algorithms were classed as moderately important, and those that appeared in just two of the algorithms were classed as low in importance. This analysis was performed using the rWeka package in R (19), which is an R interface for the WEKA machine learning statistical software package (20).

#### Parameter optimization

In order to establish the parameters that were optimized for each attribute in the data, a sub-vector of parameters was initialized for each attribute. This vector was a sequence of 100 equally distributed parameters starting from the minimum value for each respective attribute in the data and ending at the maximum values. Following this, a set of logical attributes were generated that corresponded to each athlete in the sample being either over or under each respective parameter in the vector. For instance, for the attribute “*number of sports sampled at age 12*” with a respective parameter of “*2.5*”, the new logical attribute would become “*number of sports sampled at age 12 over 2.5”*, which would thus allow for the expression of a simple logical statement about the dataset. Moreover, each athlete was assigned a 1 if their value for the given attribute was above the parameter specified in the vector, and 0 if their value was below this parameter. Consequently, a total of 100 logical attributes were generated for each original attribute that appeared in the data.

For each logical attribute, odds ratios, along with respective *p*-values, were generated for both the top-down and bottom-up samples. The lowest parameter for which the odds ratio *p*-values were < 0.05 in both top-down and bottom-up samples were selected as the optimized parameter and put forward as the final logical attribute. For any cases in which both *p*-values were not < 0.05, then the lowest parameter was selected for any logical attribute that had at least one *p*-value < 0.05, or the lowest parameter of the entire range for any attributes which did not have any significant *p*-values.

#### Odds ratio calculation

**Figure 1:**
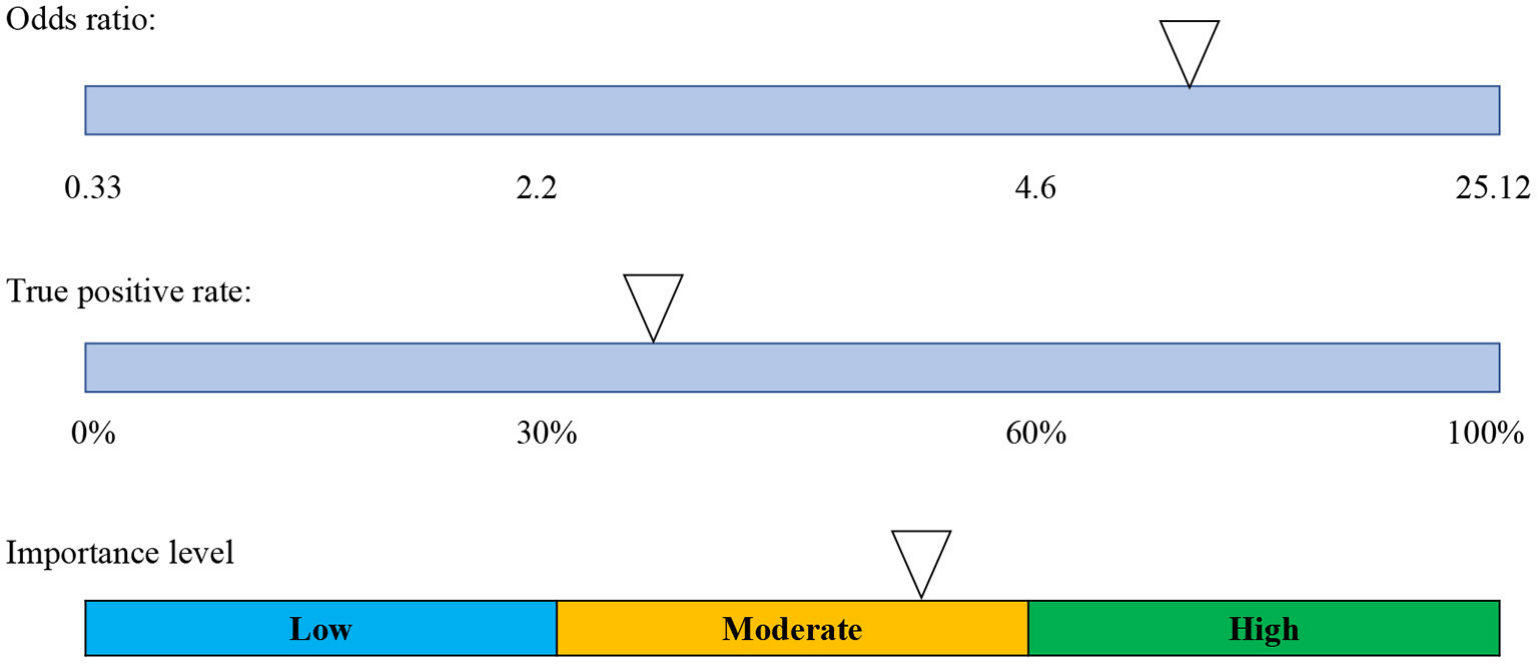
A visual representation of the method used to determine the level of importance of a significant logical attribute. As can be seen, the importance level was determined by finding the midway point between the respective points (represented as arrows) on the odds ratio size and true positive rate scales. An attribute was thus considered highly important if the odds ratio was above 4.6 whilst simultaneously having a true positive rate above 60%.

Odds ratios were calculated for each logical attribute in the data. Odds ratios were adjusted for small samples using the small method, and *p*-values and confidence intervals were calculated using the Fischer's exact method. A logical rule was considered a discriminator for high performance if the *p*-values for the associated odds ratio was below 0.05. For any significant logical attribute, a level of importance was determined by combining the size of the odds ratio with the prevalence of occurrence in the respective high performing sample [also known as the true positive rate (TPR)]. This was achieved by determining the midway point between the odds ratio and the TPR on the respective scales (see Figure 1). The cut off values for the odds ratio scale in the figure were selected as the 0th, 33rd, 66th, and 100th percentiles of odds ratios in the dataset, respectively. The rationale behind the combination of these two scales in determining the level of importance are such that both the odds ratio score and the rate of occurrence in the high-performance group need to be fairly high in order for an attribute to be deemed as important. Conversely, for any logical rules that did not appear as discriminators, commonalities were determined on the basis that: (a) a high proportion of each group (~60% or more) met the condition; and (b) the logical attribute contained theoretical relevance as a commonality. These commonalities amongst the sample could thus be identified as a necessary baseline condition to become involved in weightlifting to begin with.

## Results

### Machine learning

A summary model was produced using a Bayesian pattern recognition analysis to determine the final model of features which was to be put forward to classification. To create the model, feature selection was performed on all normalized attributes (such that the minimum and maximum values for each attribute was represented as 0 and 1, respectively) in the data. This approach enables us to identify the most pertinent attributes which determine the likelihood of an athlete “making it” as an Olympic weightlifter. This process determined a model of 11 multidisciplinary features which were grouped into three distinct levels of importance based on their appearance in the top 20 features of all four, any three, or any two of the feature selection algorithms, respectively. Table 2 shows the features in this model.

**Table 2:** The list of attributes selected for the summary model, along with their rating of importance and direction of influence on weightlifting performance.

**Attribute**	**Importance level**	**Direction of influence**	
		**“Low” performance**	**“High” performance**
**Demographics and family:**
1. School main place for sport participation (6–12 years)	Very important	–	+
**Psychosocial**			
2. Perfectionism: Doubts about actions	Fairly important	+	–
**Sport participation history and weightlifting specific involvement**			
3. Flexibility/mobility training at age 11	Important	–	+
4. Flexibility/mobility training by age 14	Very important	–	+
**Practice activities**			
5. Proportion of information received as demonstration at T1	Important	–	+
6. Proportion of extrinsic feedback by T1	Important	+	–
7. Volume of flexibility/mobility practice by T1	Important	–	+
8. Volume of snatch whole practice by T1	Fairly important	–	+
9. Change in proportion of information received as video feedback information between T1 and T2	Fairly important	–	+

**Figure 2:**
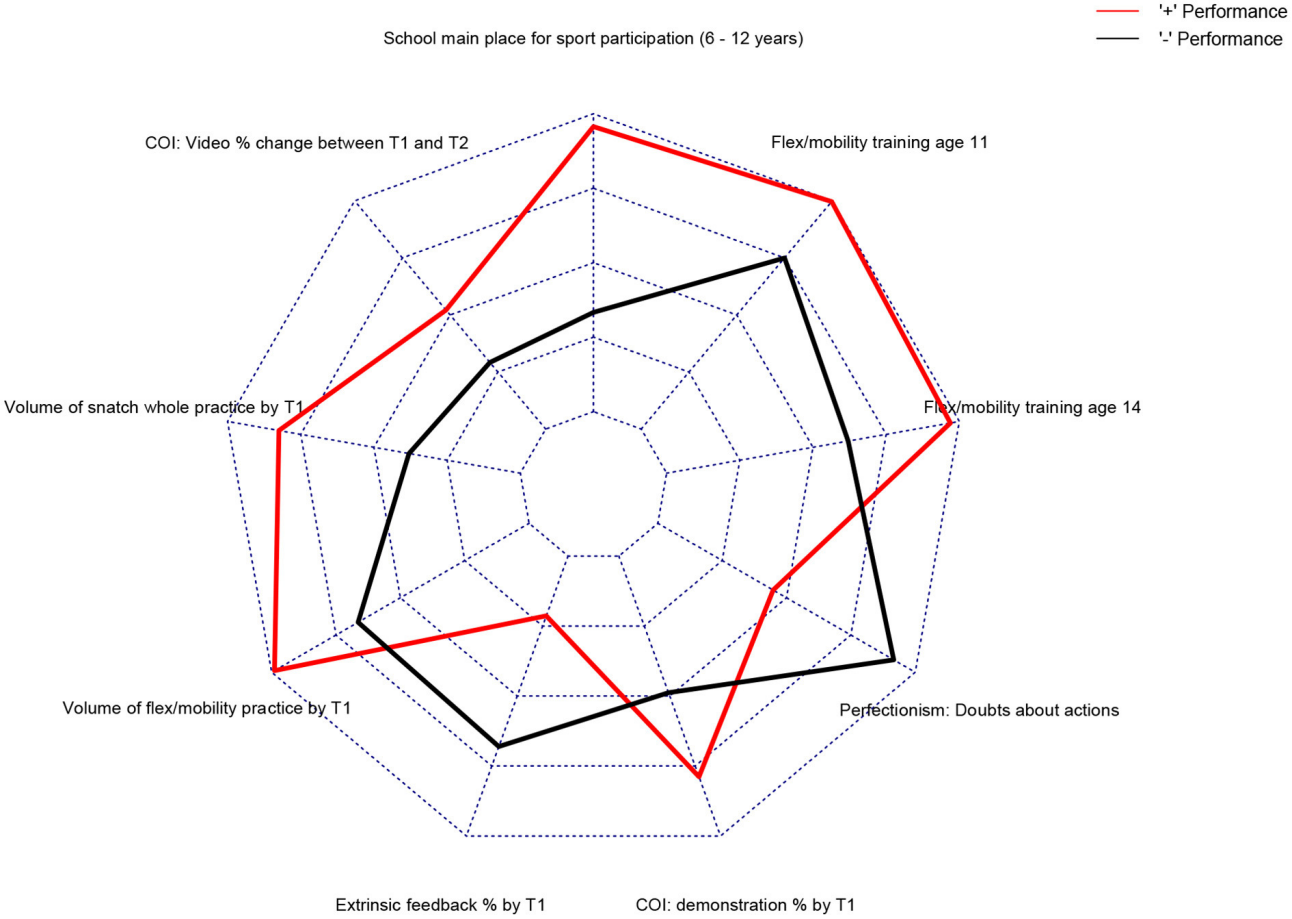
A radar plot depicting the normalized means for both performance groups on each attribute in the summary model. Attributes are placed clockwise by order of chronological occurrence starting from participation in sport at school.

For the next step in the analysis, the model's ability to differentiate the performance groups was assessed against four different classification algorithms. For this step, four commonly used classification algorithms were used, namely the Naïve Bayes [cf. (21)], J48 decision tree [cf. (22)], Support Vector Machine [SMO; cf. (23)], and K-nearest neighbors (24). This classification process was performed iteratively using a leave one out cross-validation procedure to minimize overfitting the findings to the data and thus preserving the generalizability of the model. The result of this classification process can be seen in Table 3. The model was able to differentiate 91% of the sample across all four classification algorithms successfully. An average area under the curve (AUC) of 0.87 indicates that this model contains a moderate to high predictive power (25). The final model with normalized group means is shown as a radar plot in Figure 2. As is shown, clear separation exists between the groups on each attribute within the model.

**Table 3:** Summary statistics for all four classification algorithms.

**Classifier**	**Accuracy**	**Sensitivity**	**Specificity**	**Area under ROC curve**
Naïve Bayes	96.55%	1.000	0.833	0.986
Support vector machine	96.55%	1.000	0.833	0.916
J48 decision tree	82.8%	0.913	0.500	0.836
K-Nearest neighbor	86.2%	0.957	0.500	0.728
All classifiers	90.5%	0.967	0.667	0.867

Accuracy = Correctly classified observations/total number of observations. Sensitivity = 1 – false positive rate. Specificity = 1 – false negative rate. Area under ROC curve is a measure of model's ability to correctly distinguish the two groups.

ROC, Receiver operating characteristic.

### Discriminating attributes

The logical rules for each attribute included in the analysis for demographics and family, anthropometrics, psychosocial characteristics, sport history, and microstructure of practice are presented in Tables 4–7, Supplementary material 1 along with odds ratios, which can be used to determine the likelihood of an athlete reaching higher performance status based on individual attributes. For example, if we take “demographics and family” athletes were “14.67” times more likely to become classified as high performing if their School formed the primary location for participation in sport between the ages of 6 and 12 years.

**Table 4:** Logical attributes for all discriminative features within demographics and familial sport participation.

**Attribute**	**Low performing**	**High performing**	**OR (95% CI)**	**Importance**
**Homeplace throughout development**				
Population of longest residing homeplace between 6 and 12 years >11,369	6/23 (26.1%)	5/6 (83.3%)	6.07 (1.31–74.22)	High
**Schooling**				
School main place for sport participation between 6 and 12 years	1/23 (4.3%)	4/6 (66.7%)	14.67 (2.81–259.57)	High

**Table 5:** Logical attributes for all discriminative features within anthropometrics and physiology.

**Attribute**	**Low performing**	**High performing**	**OR (95% CI)**	**Importance**
**Between T1 and T2:**				
Difference in height T1–T2 >1.5 cm above norm	2/23 (8.7%)	4/6 (66.7%)	9.33 (2.04–117.42)	High
**Body segments:**				
**By T1:**				
Tibia length > 3.79 cm above norm	0/23 (0%)	2/6 (33.3%)	9.2 (1.06–640.23)	Moderate
Total Arm to height ratio above norm	7/23 (30.4%)	6/6 (100%)	12 (1.42–576.26)	High
**Between T1 and T2:**				
Difference in torso length between T1 and T2 > 1.54 cm above norm	1/23 (4.3%)	3/6 (50%)	8.25 (1.63–138.06)	Moderate
Difference in tibia length between T1 and T2 > 0.16 cm above norm	6/23 (26.1%)	5/6 (83.3%)	6.07 (1.31–74.22)	High
Difference in total arm to height ratio between T1 and T2 > 0.03 cm below norm	0/23 (0%)	2/6 (33.3%)	9.2 (1.06–640.23)	Moderate
**Stretch shortening cycle utilization:**				
**By T1:**				
Duncan estimate for countermovement jump peak power > 225.28 W above norm	6/23 (26.1%)	5/6 (83.3%)	6.07 (1.31–74.22)	High
Maximum standing broad jump distance > 11.08 cm above norm	4/23 (17.4%)	4/6 (66.7%)	5.07 (1.22–49.85)	High
**By T2:**				
Sayers estimate for countermovement jump peak power > 317.21 W above norm	6/23 (26.1%)	5/6 (83.3%)	6.07 (1.31–74.22)	High
Duncan estimate for countermovement jump peak power > 232.77 W above norm	6/23 (26.1%)	5/6 (83.3%)	6.07 (1.31–74.22)	High
Maximum countermovement jump height > 1.48 cm above norm	6/23 (26.1%)	5/6 (83.3%)	6.07 (1.31–74.22)	High
Maximum squat jump height > 12.72 cm above norm	0/23 (0%)	2/6 (33.3%)	9.2 (1.06–640.23)	Moderate
**Skeletal muscle strength:**				
**By T1:**				
Back Squat to body mass ratio > 0.67 above norm	1/23 (4.3%)	3/6 (50%)	8.25 (1.63–138.06)	Moderate
Front Squat to body mass ratio > 0.03 above norm	8/23 (34.8%)	6/6 (100%)	10 (1.19–474.06)	High
**By T2:**				
Back Squat body mass ratio > 1.02 above norm	0/23 (0%)	2/6 (33.3%)	9.2 (1.06–640.23)	Moderate
Front Squat body mass ratio > 0.48 above norm	2/23 (8.7%)	3/6 (50%)	5.25 (1.19–62.13)	Moderate
**Between T1 and T2:**				
Difference in back squat to body mass ratio between T1 and T2 > 0.01 above norm	12/23 (52.2%)	0/6 (0%)	0 (0–1.4)	Low
**Mobility/trunk stability:**				
**By T1:**				
OHS torso > 66.71 degrees	0/23 (0%)	2/6 (33.3%)	9.2 (1.06–640.23)	Moderate

**Table 6:** Logical attributes for all discriminative features within psychosocial characteristics.

**Attribute**	**Low performing**	**High performing**	**OR (95% CI)**	**Importance**
**Athlete behaviors and attitudes toward training and competition**				
**Achievement motivation:**				
Mastery approach at least 6 (out of 7)	1/23 (4.3%)	3/6 (50%)	8.25 (1.63–138.06)	Moderate
Mastery avoidance at least 4 (out of 7)	21/23 (91.3%)	2/6 (33.3%)	0.04 (0.01–0.49)	High
Performance approach at least 6 (out of 7)	4/23 (17.4%)	4/6 (66.7%)	5.07 (1.22–49.85)	High
**Athlete behaviors and attitudes:**				
Commitment to training > 4.7	10/23 (43.5%)	6/6 (100%)	7.09 (0.84–331.5)	High
Total preparation for competition > 5.15	6/23 (26.1%)	3/6 (50%)	1.82 (0.48–15.24)	
Relative importance of sport > 3.55	10/23 (43.5%)	6/6 (100%)	7.09 (0.84–331.5)	High
Harmonious passion > 5.72	2/23 (8.7%)	5/6 (83.3%)	17.5 (3.39–293.37)	High
Obsessive passion > 4.63	4/23 (17.4%)	5/6 (83.3%)	9.5 (1.99–126.68)	High
**Athlete personality**				
Conscientiousness > 5.81	5/23 (21.7%)	5/6 (83.3%)	7.5 (1.6–94.94)	High
Openness to experience > 6.59	0/23 (0%)	2/6 (33.3%)	9.2 (1.06–640.23)	Moderate
Extraversion > 5.21	9/23 (39.1%)	6/6 (100%)	8.4 (1–394.78)	High
**Perfectionism:**				
Doubts about actions > 2.35	22/23 (95.7%)	0/6 (0%)	0 (0–0.14)	High
Organization > 5.61	2/23 (8.7%)	5/6 (83.3%)	17.5 (3.39–293.37)	High

**Table 7:** Logical attributes for all discriminative features within sport participation through development.

**Attribute**	**Low performing**	**High performing**	**OR (95% CI)**	**Importance**
**Weightlifting related involvement:**				
**Flexibility/mobility training (h per week) at:**				
Age 10 > 0.15 h	0/23 (0%)	2/6 (33.3%)	9.2 (1.06–640.23)	Moderate
Age 11 > 0.17 h	0/23 (0%)	2/6 (33.3%)	9.2 (1.06–640.23)	Moderate
Age 12 > 0.88 h	0/23 (0%)	2/6 (33.3%)	9.2 (1.06–640.23)	Moderate
Age 13 > 0.93 h	0/23 (0%)	2/6 (33.3%)	9.2 (1.06–640.23)	Moderate
Age 14 > 1.66 h	0/23 (0%)	4/6 (66.7%)	30.67 (3.45–2,074.36)	High
Age 15 > 1.85 h	0/23 (0%)	3/6 (50%)	17.25 (1.98–1,117.65)	High
**Strength and conditioning training (h per week):**				
Age 9 > 0.39 h	0/23 (0%)	2/6 (33.3%)	9.2 (1.06–640.23)	Moderate
Age 10 > 0.49 h	0/23 (0%)	2/6 (33.3%)	9.2 (1.06–640.23)	Moderate
Age 15 > 2.13 h	1/23 (4.3%)	3/6 (50%)	8.25 (1.63–138.06)	Moderate
**Total combined flex/mob, strength and conditioning, and weightlifting specific practice (h per week):**				
Age 13 > 0.78 h	17/23 (73.9%)	2/6 (33.3%)	0.06 (0.01–0.76)	Low
Age 15 > 9.58 h	3/23 (13%)	5/6 (83.3%)	12.5 (2.55–181.04)	High
**Cumulative practice volumes by T1:**				
Flexibility/mobility practice > 255.79 h	0/23 (0%)	3/6 (50%)	17.25 (1.98–1,117.65)	High
Strength and conditioning training > 936.24 h	0/23 (0%)	3/6 (50%)	17.25 (1.98–1,117.65)	High
Weightlifting specific practice > 657.18 h	4/23 (17.4%)	5/6 (83.3%)	9.5 (1.99–126.68)	High
Number of competitions > 8	8/23 (34.8%)	6/6 (100%)	10 (1.19–474.06)	High
Competition time > 43.08 h	4/23 (17.4%)	5/6 (83.3%)	9.5 (1.99–126.68)	High
**Cumulative practice volumes by T2:**				
Flexibility/mobility practice > 195.3 h	1/23 (4.3%)	4/6 (66.7%)	14.67 (2.81–259.57)	High
Strength and conditioning training > 603.17 h	1/23 (4.3%)	3/6 (50%)	8.25 (1.63–138.06)	High
Weightlifting specific practice > 1,527.54 h	2/23 (8.7%)	4/6 (66.7%)	9.33 (2.04–117.42)	High
Number of competitions > 10	8/23 (34.8%)	6/6 (100%)	10 (1.19–474.06)	High
Competition time > 53.49 h	3/23 (13%)	5/6 (83.3%)	12.5 (2.55–181.04)	High
**Cumulative practice volumes between T1 and T2:**				
Flexibility/mobility practice > 29.18 h	5/23 (21.7%)	6/6 (100%)	18 (2.11–904.9)	High
Weightlifting specific practice > 392.28 h	2/23 (8.7%)	3/6 (50%)	5.25 (1.19–62.13)	High

### Demographics and family

Two attributes were identified as discriminatory features of expertise development: population of longest residing homeplace (with a higher proportion of higher performing athletes living in a town with a population >11,368) and school as the primary location for participation in sport through the development years.

### Physiological profile

#### Anthropometrics

Differences in the anthropometrics of the two groups were observed at a combination of the cross-sectional (by T1), and longitudinal (between T1 and T2) levels. Higher proportions of higher performing athletes had tibia lengths more than 3.8 cm longer than the expected values for their respective age and gender, and total arm length to height ratios above that expected. Between T1 and T2, a higher proportion of high performing athletes recorded growth in height more than 1.5 cm above the normative value for their age and gender. This was accompanied by greater respective increases in both the torso length (>1.54 cm) and tibia length (>0.16 cm) for this group.

#### Stretch shortening cycle utilization

Differences in the diagnostic measurements for stretch shortening cycle utilization were observed between the groups at the cross-sectional level (by both T1 and T2) only. At T1, there was a greater proportion of high performing athletes who had achieved a standing broad jump distance more than 11 cm above their expected value for achieve and gender. Similar observations were also observed for the peak power estimate of the countermovement jump. By T2, discrimination occurred between the groups for the countermovement jump height and squat jump height, as well as estimates for CMJ peak power.

#### Maximum dynamic strength

An important discriminator in the physiological dataset was the back and front squat to body mass ratio. This attribute produced significant odds ratios at both the cross-sectional (both T1 and T2) and longitudinal (between T1 and T2) level. There was a greater proportion of high performing athletes reported having both front squat and back squat to body mass ratios that were above the normative ratio for age and gender by T1 and by T2. These results are unsurprising, given the high correlation between maximum dynamic strength and weightlifting performance, particularly in relation to the back squat [*r* = 0.86; (26)]. Attainment of a maximum back squat to body mass ratio 0.7 units above the expected, increased the likelihood of high performance by ~8-fold. However, between T1 and T2, the opposite trend emerged in the back squat to body mass ratio data. This is likely a result of the higher performing group having a higher back squat to body mass ratio to begin with, meaning that further improvements beyond that expected for age and gender were less likely to occur.

#### Trunk stability

Odds ratios for this attribute were significant at the cross-sectional level (T1) only and suggest that the ability to achieve a position in the squat which allows the angle of the torso to be open enough to support the position of the barbell will support a balanced weight distribution through the squat.

### Psychosocial profiles

#### Attitude toward training and competition

There were five attributes identified as being indicative of high-performance: mastery approach and avoidance, performance approach, commitment to training, and relative importance of sport. Mastery approach was positively associated with attainment of high performance and conversely, mastery avoidance (referring to the motivation for training that is driven by the avoidance of self-referenced incompetence) seemed indicative of lower performance. Higher performing athletes were also more likely to report a stronger performance approach; an ego-oriented motivation construct whereby individuals high in this construct tend to be highly motivated to demonstrate competence by outperforming others. Furthermore, the higher performing group reported higher levels of commitment to training, which aimed to target the athlete's degree of motivation toward attending and completing all the necessary training for competitions by fitting as much training into the week as possible as well as trying to make training sessions as productive as possible. Similarly, the high-performance group reported greater relative importance of sport. A high relative importance of sport suggests that an individual perceives their involvement in sport as more important than other life choices, such as personal relationships and other potential life choices.

#### Athlete personality

Three of the “big five” personality traits; conscientiousness, extraversion, and openness to experience differentiated between our two athlete groups. Athletes scoring above 5.8 on conscientiousness were 7.5 times more likely to attain high performance status. This attribute describes the ability to control impulse-related behaviors in order to preserve task- and goal-directed behavior (27). Similarly, athletes scoring above 5.21 and 6.29 for extraversion and openness to experience were 8.4 and 9.2 times more likely to achieve high performance, respectively. Outside of the big five, organization, doubts about actions, and harmonious and obsessive passion were identified as determinants of expertise. Organization stems from an adaptive form of perfectionism and is described as tendencies to establish and implement routines or plans that guide behavior prior to and during competition. This finding confirms the importance of athletes to be meticulous in their preparation for competition, particularly with regards to pre-planned routines. Another construct of perfectionism that appeared to be a discriminatory feature was doubts about actions. This presented as an undesired characteristic and seems somewhat intuitive in the context of weightlifting, being that the margin for error during competitions are small, and as such any overriding concerns or doubts about one's own action could result in negative performance consequences. Finally, harmonious and obsessive passion appeared to be a dominant feature in the high-performance sample.

### Sporting history and weightlifting specific involvement

Whilst the engagement in high volumes of weightlifting related training throughout the sampling years was not a prerequisite for weightlifting participation, a degree of involvement from an early age did appear to be a discriminator. This incorporated more generalized strength and conditioning work as well as flexibility and mobility from as early as 9–10 years. There was also evidence for a progressive increase in volume of flexibility and mobility training with age, up to 30 min of flexibility and mobility training per week by the ages of 12 and 13, with this increasing to at least 50 min per week by the age of 12, and more than 1 h 45 min by age 15. Early exposure to weightlifting related training was also reflected in the total volume of practice in each of the weightlifting related domains the high performing groups had accumulated up until the beginning of the study as well between T1 and T2. High performing athletes completed a minimum of 44 min of flexibility and mobility training per week.

For weightlifting specific involvement, evidence existed at both the cross-sectional and longitudinal level, that the total volume of practice was an important discriminator of performance. High performing athletes completed approximately 9 h 50 min of weightlifting specific practice per week. The high performing group were also more exposed to competitions prior to baseline testing. Competition time included exposure to the competition environment itself, such as weighing in on the morning of a competition, managing the food intake between the weigh-in and competition time, warming up for the competition, as well as competing in the competition itself.

An interesting finding was observed in relation to the combined volume of weightlifting related and weightlifting specific practice between the ages of 13–15. Specifically, at 13 years of age, a significantly large proportion of the low performing athletes were completing at least 50 min of weightlifting specific or related practice per week, whilst only two of the high performing athletes were completing this volume. The relationship had reversed by age 15, with five of the six high performing athletes completing volumes of 9.58 h per week, and only three of the 23 low performing athletes reporting this. This finding is potentially indicative of a transition between sampling to specialization in the high performing group, with the onset of high-volume training occurring at 15 years. On the other hand, the low performing group did not demonstrate this transition into investment, as a large proportion of this group did not demonstrate increases in training volumes by this age.

### Microstructure of practice (see Supplementary material for full statistical detail)

#### Volume of mental skills training

The volume of mental skills training that was reported appeared as a discriminatory feature in this sample. Mental skills training referred to the amount of time during a typical week which was spent mentally rehearsing their own performance routines (usually through imagery) or reflecting on past training and competition experiences. A higher proportion of high performing athletes reported undergoing mental skills training for at least 14 h per week (i.e., equivalent to 2 h per day) at both T1 and T2, whilst only one of the 23 high performing athletes reported completing this amount. It is likely that the greater volume of mental skills training reported in the high-performance sample promoted a higher level of mental preparation in these athletes both in relation to training and competition.

#### Vicarious experiences

A higher proportion of high performing athletes reported completing at least 4 h 15 min of vicarious experiences per week by T1. This incorporated time spent observing other athletes prepare for and compete in training and competition. Given that a baseline amount of vicarious experiences of 53 min was established as a commonality amongst the cohort by T2, it is likely that a higher volume of vicarious experiences would be required in order to achieve high performance.

#### Conveying of information

The proportion of the types of information that is conveyed to the athlete was a discriminating feature of the dataset at both the cross-sectional and longitudinal level. Interestingly, higher performing athletes appeared to higher proportions of information *via* non-verbal formats, e.g., demonstrations and videos.

#### Whole vs. part practice

The proportions of whole vs. part practice in the clean and jerk, as well as the volumes of whole practice for the snatch, whole and part practice for the clean and jerk were identified as discriminating features. Higher performing athletes reported higher proportions of whole practice for the clean and jerk at T1 and T2 and between T1 and T2. Athletes were doing >1 h 15 min per week. Similarly, these athletes also reported completing higher volumes of snatch practice by T1 and higher volumes of whole snatch practice between T1 and T2.

#### Constant vs. varied practice

Another important discriminating feature in this sample appeared to be the proportion of time spent practicing with an environment that induced variable practice conditions. Higher performing athletes reported completing higher proportions of varied practice (>21% of overall practice) and conversely, lower performing athletes reported higher proportions of constant practice by T1. By T2, volumes of varied practice increased further, equating to ~3 h 25 min weekly.

#### Specificity of practice

The proportions of practice conditions that matched the specific demands of competition conditions, as well as practice volumes, also emerged as discriminating features. Higher performing athletes reported greater proportions of practice with anxiety specific conditions (>31% by T1). Between T1 and T2, most high performing athletes were completing ~2 h 45 min of anxiety specific practice per week and reported higher levels of context specificity.

#### Focus of attention

Although no differences in the proportions of attentional foci adopted during practice were observed between the groups, the groups differed on the volumes of practice using both internal (i.e., body related) and external (i.e., outside of body related) attentional foci, with higher performing athletes reporting greater volumes.

#### Sources of feedback

The proportions of externally vs. internally derived feedback also positively discriminated between the groups. Evidence for this finding was solely derived from cross sectional observations with higher performing athletes reporting higher proportions of feedback from inherent (intrinsic) sources and lower performing athletes reporting at least 81% of feedback was primarily derived from augmented sources such as the coach.

#### Prescriptive vs. constraints-based coaching

A further key discriminating feature was the proportions and volumes of prescriptive and constraints-based coaching. Lower performing athletes reported higher proportions of prescriptive coaching (by T1 and T2), whereas higher performing athletes reported higher proportions of constraints led coaching (>30% by T2). Higher performing athletes were completing ~1 h 45 min of this type of practice per week between T1 and T2.

## Discussion

The rationale behind the current study was to identify important antecedents of expertise development in a sport specific (Olympic weightlifting) context. Findings were informed by prospective, multidisciplinary, and longitudinal data to ensure trustworthiness and generalizability of findings. Sophisticated machine learning techniques were adopted to better operationalize the complexities and dynamic nature of talent. Data pertaining to (a) demographics and family; (b) anthropometrics and physiology; (c) psychosocial factors; (d) sport participation history; and (e) weightlifting specific practice activities were able to differentiate between two groups of athletes: classified as being either high or low performing. For all the attributes presented in our summary model (Figure 2), it is important to note that it is the combination of potential interactions between these attributes that discriminate between high and low performance rather than any standalone attribute. Attributes may well be interacting in any number of complex ways when discriminating between athletes and these are likely to be beyond even 3 dimensional in nature. Our discussion attempts to make sense of these interactions based on the study's theoretical framework. In addition, we presented and discussed the odds ratios for individual attributes given that we had so many in study (i.e., 648). This allowed for both the main machine learning model and the most important and potentially important individual attributes from each of the five subthemes to be explored and discussed from a broad multidisciplinary perspective.

Differences between the performance groups were observed within each domain of expertise development. Findings demonstrate that the development of elite performance is indeed a complex multidimensional construct. Specifically, the characterization of high performance in weightlifting emerges from a specific set of antecedents, fostered by early exposure to environments that encourage the emergence of desired motivational and physiological traits. These are then honed/fine-tuned through extensive exposure to conditions of practice that promote robust performance in competition. These antecedents take the form of living in a homeplace throughout one's development which has the appropriate infrastructure and opportunities for early sport participation (from 6 years), particularly *via* schooling. Early sport participation should include activities which promote flexibility and mobility training, as well as general functional conditioning activities, from 9 years of age. Then, likely as a result of having parents whole also participate in sport, these individuals are encouraged to participate in weightlifting from around the age of 13–14 and begin investing in their weightlifting development, perhaps as a result of containing the appropriate anthropometric profile and demonstrating considerable strength and power adaptions, from around 15 years. Additionally, perhaps as a reflection of their conscientious, open-minded, and extravertive personalities, they develop a strong passion for weightlifting which places their relative importance of the sport highly in comparison with other commitments. This manifests itself as a strong commitment to training, an approach to training that is focused on the attainment of both an absolute (i.e., an objective) and relative (i.e., a peer-related) standard of competence, as well as a high degree of organization in preparation for competition that increases their sense of self-confidence and reduces any sense of doubt about their own actions. These behaviors may also be gathered implicitly from learning vicariously through more experienced lifters, as well as through means of mental reinforcement outside of the training environment. In training, they engage in extensive volumes of weightlifting specific practice in a setting that offers a constant and predictable environment, as well as in training settings that offers variation to the athlete, such as in a different club. They practice the execution of the snatch and clean and jerk both as whole movements, as well as in assistance exercises which enable practice of each movement to be broken down into parts. They are sure, however, to sustain an optimal proportion of the technical practice for both lifts as they are intended for competition (as whole movements). Throughout their training, they initially receive a high proportion of information from their coach through verbal instruction and physical demonstrations but are encouraged to progressively use more video information sources the more experienced they become. Feedback about their performance is mainly *via* extrinsic means, although they tend to produce higher proportions of feedback from their own sensory sources with experience. This feedback is likely guided by mastery and performance approach motivation. Finally, in the lead up to a competition, their training begins to closely meet the demands of competition, both in relation to context and the perception of anxious states, which allows them to optimally transfer to the competitive stage. Moreover, whilst this combination may support generalized theoretical concepts, such as early sport sampling and extensive deliberate practice, the novelty in these findings lie in the notion that the holistic profile reported thus far is indeed specific to the sport of Olympic weightlifting.

### Demographics and family sport participation

Data suggested that Olympic weightlifting participation in Wales tends to occur in more densely populated (i.e., less rural) communities. This is consistent with literature emphasizing the role of the community in talent development^1^. As well as having enhanced infrastructure, clubs also tended to provide a service to the public. Furthermore, demographic data supports the notion that sport participation during schooling within the sampling years is an important feature in the development of high performance. Given that most of the athletes in the study didn't begin weightlifting training until 12 years onwards, this enabled greater opportunities for sampling, which may have helped foster the motivational and physiological characteristics necessary for high performance in weightlifting. Policy makers should not underestimate the value of offering adequate curricular and extra-curricular sporting opportunities. Findings also revealed a clear parental influence, particularly from a father figure, on initial engagement in the sport, consistent with literature reporting parental influences on child sport participation (28).

### Physiological characteristics

Anthropometric data highlighted the importance of longer body segment lengths as a facilitator of weightlifting performance, particularly in the lower extremities of the body. This is likely due to the mechanical advantage that longer limb lengths place on the biological lever systems of the body and is in accordance with findings reported by Musser et al. (29), who observed that thigh length in female athletes (53 kg class) produced less horizontal displacement of the barbell in the second pull of the snatch. Longer tibia lengths facilitate a more upright position in the overhead squat, and subsequently more even weight distribution in the receive and recovery positions of the snatch. Not surprisingly, there were also clear differences in the explosive power and maximum dynamic strength profiles of the two groups. This highlights the role of stretch cycling utilization and lower body power production for weightlifting performance and is in line with Fry et al. (30) who reported CMJ height to be a discriminator of weightlifting performance. Noteworthy findings were also reported in the position of the trunk during the overhead squat, which could serve as a proxy indicator of the finish position of the snatch. The ability to achieve a position in the squat which allows the angle of the torso to be open enough to support the position of the barbell better affords a balanced weight distribution through the squat. This could also be linked to longer tibia lengths and supports Fry et al. (30) who reported larger angles of the torso in an elite junior sample. We would recommend practitioners streamline power testing toward the lower extremities, e.g., CMJ and squat jump, combined with regular assessment of the torso position during the squat lift in the development of weightlifting athletes.

### Psychosocial profiles

Discriminative attributes of performance in relation to personality, included three of the big five: conscientiousness, extraversion, and openness to experience, in addition to some features of perfectionism: organization and doubts about actions. Higher levels of conscientiousness have been associated with quality of preparation (31) and has also been linked with harmonious passion (32), an association attributed to competence (33). Extraversion is in line with the “individual” nature of the sport, and refers to the tendency to attain feelings of positive affect or gratitude from outside of oneself (34). It has been found to be positively associated with harmonious passion, a further discriminator, and result in more positive emotions (32). Openness describes the breadth, depth and complexity of an individual's mental and experiential life (35). It has been positively, associated with sensation seeking (36), which is often accompanied by heightened risk taking (37). Given the intense nature of weightlifting, particularly during competition, athletes could be more attracted to engage in training for the purposes of sensation seeking in competition.

Higher levels of organization, confirms the importance of meticulous preparation for competition and supports previous findings that adaptive forms of perfectionism, can lead to performance benefits (38). Conversely, higher levels of doubts about actions, a maladaptive form of perfectionism, prevalent in the lower performing athletes, is in accordance with the notion that maladaptive forms of perfectionism are detrimental to high performance and can result in anxiety (39) and unhelpful attitudes surrounding attainment of self-confidence from uncontrollable sources (40).

With regards to attitudes toward training and competition, discriminative features were identified as both forms of mastery-oriented achievement motivation (i.e., approach and avoidance), performance approach achievement motivation, harmonious and obsessive passion, commitment to training, and a high relative importance of sport. Mastery approach describes the striving for attainment of competence at a task that is based on a self-referenced standard (41). Individuals high in this trait are suggested to demonstrate adaptive achievement behaviors, which leads to a myriad of positive outcomes such as increased intrinsic motivation (42), positive evaluations of competence, reduced state anxiety (43), and absorption in the task (44).

The finding that performance approach was associated with high performance in weightlifting is somewhat unsurprising. Given that the fundamental premise of competition is to outperform others, one would expect high performing athletes to be motivated to train and compete for the purpose of outperforming their peers to some degree Previous findings have shown this to be associated with higher levels of performance in athletes [e.g., (45)]. The fact that both performance approach goals appeared as discriminating features highlights the importance for both task- and ego-oriented forms of achievement motivation to occur in tandem for the attainment of high performance (4). Similarly, where both forms of passion for weightlifting were discriminative of performance, this is in line with previous research investigating the influence of passion on the attainment of higher performance (46), in which this positive association was also mediated by engagement in deliberate practice.

This also directly links with the finding that the athletes reported higher levels of commitment to training and relative importance of sport. Taken together with the results for the achievement motivation constructs reported above, as well as that of the higher volumes of weightlifting specific practice reported in the high-performance group, this result suggests that athletes in the higher performing group were more committed to the development of their own performance through practice. This finding of conforms to the notion of deliberate practice as a fundamental prerequisite to the attainment of expertise (13) and provides further support for Hardy et al. (4), who identified commitment to training and a high relative importance of sport as discriminating features in the attitudes of super elite athletes when compared with their lower achieving counterparts. In summary, we would recommend practitioners incorporate psychometric tools within talent testing protocols with the primary objective of informing athlete development as opposed to selection purposes.

### Sporting history and weightlifting specific involvement

Involvement in strength and conditioning and flexibility and mobility training tended to be most common in higher performing athletes throughout their middle years of development. In relation to sport participation more generally, these findings reaffirm that early sport participation is a necessary feature of weightlifting participation, which encourages athletes to be exposed to the motivational characteristics associated with sport participation (47). This is particularly true for a sport such as weightlifting, in which the ratio of training to competition time is high relative to other Olympic sports, and as such the motivation to sustain a training for extensive periods is an important feature. This also suggests that engagement in high volumes of weightlifting specific practice from a very early age is not a prerequisite for the attainment of high performance in weightlifting. In combination with the findings for the sampling of sport, these findings further support the need for sampling outside of weightlifting to occur at an earlier age, perhaps to facilitate the development of enjoyment of sport participation and to also foster motivational characteristics for sports participation. Whilst early involvement in weightlifting specific practice did not appear to be necessary for the attainment of high performance, the findings do however suggest that investment in weightlifting should occur much sooner in high performing athletes, typically from the end of the middle years of development (i.e., from the age of 15 years). Policy makers should consider the value of facilitating opportunities for earlier investment in weightlifting, whether this be *via* extra-curricular clubs or supporting funding allocations to improve infrastructure by means of facility provision and coaching expertise.

### Microstructure of practice

Findings reported in the current study conform to the narrative of high-quality practice being a necessity for the attainment of elite sports performance. More specifically, the current findings demonstrated that (a) athletes committed to a higher volume of mental skills development and were exposed to more vicarious experiences; (b) information was predominantly conveyed to high performing athletes verbally, yet the use of video information appeared to be particularly prevalent; (c) the majority of the high performing athletes practice involved practicing the lifts as whole movement; (d) practice conditions, although predominantly constant, were more varied in high performing athletes; (e) significant proportions of practice conditions in the high performing athletes were both anxiety and context specific; (f) higher volumes of practice with both external and internal attentional foci were observed in higher performers; (g) proportionately more intrinsic feedback was reported in higher performing athletes; and (h) higher proportions and volumes of constraints based coaching were reported in higher performing athletes. Whilst no differences were reported in the proportions of practice with different attentional foci, this finding does support the notion of the accumulations of high practice volumes whilst adopting a combination of an internal and external focus of attention. When probed about the types of attention adopted, most of the athletes reported alternating their attention between internal and external focuses. This warrants further investigation and whilst it is out of line with tenets of the constrained action hypothesis (48), it may provide support for Gottwald et al. (49) who suggest that performance is optimal when the most pertinent source of afferent information for task success is congruent with focus of attention. For more proprioceptive based sports such as weightlifting, it may be that adopting a focus on movements at certain moments, may better allow for error detection and correction. The above has implications for practitioners and specifically coaching practice in Olympic weightlifting.

## Limitations of the study

There were a number of limitations associated with the study. Although a longitudinal approach was adopted, it could be argued that the data collection period was limited given the true nature of long-term athlete development (14). Whilst a strength of the study, the nature of machine learning, and goal to identify features that best distinguish between two classes of objects, meant that athletes were classified into a rather binary vision of performance (i.e., high or low performing) where the reality of high performance is arguably less black and white. However, the performance criteria were agreed as a direct result of consultation with the sport's governing body, since pathway selection and progression are often based around athlete performance at a single time-point, e.g., competition results rather than a subjective coach view of longitudinal performance and/or potential. Furthermore, the analytical approach meant that discriminating attributes can only be truly understood in the context of the other attributes investigated. Whilst we have tried to discuss potential interactions based on our theoretical framework, these could be interpreted in different ways. Finally, in order to capture the truly holistic nature of athlete development, there was a need to capture a particularly large number of attributes across a multitude of talent development areas (demographics, psychosocial, sporting milestones, practice, and training activities), which resulted in 648 separate theoretically driven attributes. We tried to find a balance of collecting a broad range of data whilst not being overly intrusive to the developing athletes. This approach meant that not all of the methods for collecting these attributes are as rigorously validated as one another. For example, whilst the psychometric testing selected several items from existing validated measures, not the entire measure was used, which could bring into question overall validity of the psychosocial data. Future research might wish to address this point, but the onerous nature of data collection would likely be a hindrance to athletes and as a result compromise adherence.

## Data availability statement

The raw data supporting the conclusions of this article will be made available by the authors, without undue reservation.

## Ethics statement

The studies involving human participants were reviewed and approved by Bangor University School of Sport, Health and Exercise Sciences. Written informed consent to participate in this study was provided by the participants' legal guardian/next of kin.

## Author contributions

DA led on data collection and analysis with contribution to writing. VG led on methodological design and interpretation of findings and writing. GL contributed to methodological design and interpretation of findings and writing. All authors contributed to the article and approved the submitted version.

## Funding

This research was funded by a Knowledge Economy Skills Scholarship (KESS II), Weightlifting Wales, and Bangor University.

## Conflict of interest

Author DA is a founding partner of Talent Pathway iD. Author GL occasionally consults for Talent Pathway iD. The remaining author declares that the research was conducted in the absence of any commercial or financial relationships that could be construed as a potential conflict of interest.

## Publisher's note

All claims expressed in this article are solely those of the authors and do not necessarily represent those of their affiliated organizations, or those of the publisher, the editors and the reviewers. Any product that may be evaluated in this article, or claim that may be made by its manufacturer, is not guaranteed or endorsed by the publisher.

## Footnotes

1 https://doi.org/10.1080/02640410500432490.
