# Supplementary material for: Capturing the holistic profile of high performance Olympic weightlifting development
Source: Front Sports Act Living. 2022 Sep 30;4:986134. doi: 10.3389/fspor.2022.986134 (PMC9561401; doi:10.3389/fspor.2022.986134)
Supplement: Supplementary file 1 [file Table_1.DOCX]

**Supplementary Material**

**Logical attributes for all discriminative features within microstructure of practice**

| Attribute | Low Performing | High Performing | OR (95% CI) | Importance |
| --- | --- | --- | --- | --- |
| ***Deliberate practice vs play*** |  |  |  |  |
| **By T1:** |  |  |  |  |
| Volume of deliberate play >313.8 hrs | 0/23 (0%) | 2/6 (33.3%) | 9.2 (1.06 - 640.23) | Moderate |
| Volume of deliberate practice >1078.9 hrs | 3/23 (13%) | 4/6 (66.7%) | 6.67 (1.55 - 71.7) | Moderate |
| **By T2:** |  |  |  |  |
| Volume of deliberate practice more than 1426.51 hrs | 2/23 (8.7%) | 4/6 (66.7%) | 9.33 (2.04 - 117.42) | High |
| **Between T1 and T2:** |  |  |  |  |
| Volume of deliberate practice between T1 and T2 >297.98 hrs | 2/23 (8.7%) | 3/6 (50%) | 5.25 (1.19 - 62.13) | Moderate |
| ***Mental skills training (hours per week)*** |  |  |  |  |
| At T1 >14.2 hrs | 1/23 (4.3%) | 3/6 (50%) | 8.25 (1.63 - 138.06) | Moderate |
| At T2 >15.7 hrs | 1/23 (4.3%) | 3/6 (50%) | 8.25 (1.63 - 138.06) | Moderate |
| ***Vicarious experiences (hrs per week)*** |  |  |  |  |
| By T1 >4.1 hrs | 4/23 (17.4%) | 4/6 (66.7%) | 5.07 (1.22 - 49.85) | High |
| ***Information conveyed to the athlete*** |  |  |  |  |
| **At T1:** |  |  |  |  |
| Over 40.49% verbal information | 23/23 (100%) | 4/6 (66.7%) | 0 (0 - 0.94) | Moderate |
| Over 31.07% demonstration information | 3/23 (13%) | 5/6 (83.3%) | 12.5 (2.55 - 181.04) | High |
| **At T2:** |  |  |  |  |
| Over 50.95% verbal information | 23/23 (100%) | 1/6 (16.7%) | 0 (0 - 0.16) | Moderate |
| Over 29.69% demonstration information | 19/23 (82.6%) | 6/6 (100%) | 1.2 (0.14 - 63.58) |  |
| Over 18.08% video information | 6/23 (26.1%) | 5/6 (83.3%) | 6.07 (1.31 - 74.22) | High |
| **Between T1 and T2:** |  |  |  |  |
| Reduction in verbal information by >10 % units | 0/23 (0%) | 3/6 (50%) | 17.25 (1.98 - 1117.6) | High |
| Increase in video information by >10.3 % unit | 0/23 (0%) | 3/6 (50%) | 17.25 (1.98 - 1117.6) | High |
| ***Whole/Part Practice*** |  |  |  |  |
| **For the snatch:** |  |  |  |  |
| **At T1:** |  |  |  |  |
| Volume of snatch part practice by T1 >247.8 hrs | 4/23 (17.4%) | 5/6 (83.3%) | 9.5 (1.99 - 126.68) | High |
| Volume of snatch whole practice by T1 >265.9 hrs | 0/23 (0%) | 4/6 (66.7%) | 30.67 (3.45 - 2074.3) | High |
| **At T2:** |  |  |  |  |
| Volume of snatch part practice by T2 >313.2 hrs | 4/23 (17.4%) | 5/6 (83.3%) | 9.5 (1.99 - 126.6) | High |
| Volume of snatch whole practice by T2 >327.2 hrs | 0/23 (0%) | 4/6 (66.7%) | 30.67 (3.45 - 2074.3) | High |
| **Between T1 and T2:** |  |  |  |  |
| Volume of snatch whole practice between T1 and T2 > 69.6 | 1/23 (4.3%) | 3/6 (50%) | 8.25 (1.63 - 138.0) | High |
| **For the clean & jerk:** |  |  |  |  |
| **At T1:** |  |  |  |  |
| Proportion of clean & jerk practice as parts >49% | 23/23 (100%) | 4/6 (66.7%) | 0 (0 - 0.94) | Moderate |
| Proportion of clean & jerk practice as whole movement >51% | 0/23 (0%) | 2/6 (33.3%) | 9.2 (1.06 - 640.23) | Moderate |
| Volume of clean & jerk part practice by T1 >241.2 hrs | 4/23 (17.4%) | 5/6 (83.3%) | 9.5 (1.99 - 126.68) | Moderate |
| Volume of clean & jerk as whole practice >206.3 hrs | 0/23 (0%) | 3/6 (50%) | 17.25 (1.98 - 1117.6) | Moderate |
| **At T2:** |  |  |  |  |
| Proportion of clean & jerk practice as parts >71% | 15/23 (65.2%) | 0/6 (0%) | 0 (0 - 0.84) | Moderate |
| Volume of clean & jerk part practice >315 hrs by T2 | 4/23 (17.4%) | 5/6 (83.3%) | 9.5 (1.99 - 126.68) | High |
| Volume of clean & jerk as whole practice >132.3 hrs by T2 | 4/23 (17.4%) | 4/6 (66.7%) | 5.07 (1.22 - 49.85) | High |
| **Between T1 and T2:** |  |  |  |  |
| Volume of clean & jerk whole practice >50 hrs practice between T1 and T2 | 2/23 (8.7%) | 4/6 (66.7%) | 9.33 (2.04 - 117.42) | High |
| ***Constant vs Varied Practice*** |  |  |  |  |
| **At T1:** |  |  |  |  |
| Constant Practice proportion >86% | 17/23 (73.9%) | 1/6 (16.7%) | 0.06 (0.01 - 0.76) | Moderate |
| Varied Practice proportion >21% | 3/23 (13%) | 4/6 (66.7%) | 6.67 (1.55 - 71.7) | High |
| Volume of practice with varied practice >73.52 hours | 1/23 (4.3%) | 3/6 (50%) | 8.25 (1.63 - 138.06) | Moderate |
| **At T2:** |  |  |  |  |
| Varied Practice proportion over 12% | 6/23 (26.1%) | 5/6 (83.3%) | 6.07 (1.31 - 74.22) | Moderate |
| Volume of practice with varied practice >222.39 hrs | 1/23 (4.3%) | 4/6 (66.7%) | 14.67 (2.81 - 259.57) | Moderate |
| **Between T1 and T2:** |  |  |  |  |
| Volume of varied practice between T1 and T2 >135.62 hrs | 0/23 (0%) | 2/6 (33.3%) | 9.2 (1.06 - 640.23) | Moderate |
| ***Specificity of Practice*** |  |  |  |  |
| **Anxiety Specificity:** |  |  |  |  |
| **By T1:** |  |  |  |  |
| Proportion of overall practice >31.08% | 1/23 (4.3%) | 3/6 (50%) | 8.25 (1.63 - 138.06) | Moderate |
| Volume of Anxiety Specificity training >443.9 hrs | 0/23 (0%) | 3/6 (50%) | 17.25 (1.98 - 1117.65) | High |
| **By T2:** |  |  |  |  |
| Volume of Anxiety Specificity T2 >556.77 | 0/23 (0%) | 3/6 (50%) | 17.25 (1.98 - 1117.65) | High |
| **Between T1 and T2:** |  |  |  |  |
| Accumulated a volume of anxiety specificity practice by >112 hrs between T1 and T2 | 1/23 (4.3%) | 4/6 (66.7%) | 14.67 (2.81 - 259.57) | High |
| **Context Specificity:** |  |  |  |  |
| **By T1:** |  |  |  |  |
| Volume of context specificity training >62.06 hrs | 4/23 (17.4%) | 4/6 (66.7%) | 5.07 (1.22 - 49.85) | High |
| **By T2:** |  |  |  |  |
| Volume of context specificity training >1071.64 hrs by T2 | 2/23 (8.7%) | 3/6 (50%) | 5.25 (1.19 - 62.13) | High |
| ***Focus of Attention*** |  |  |  |  |
| **By T1:** |  |  |  |  |
| Volume of practice with internal focus of attention >281.26 hrs by T1 | 4/23 (17.4%) | 5/6 (83.3%) | 9.5 (1.99 - 126.68) | High |
| Volume of practice with external focus of attention >346.04 hrs by T1 | 2/23 (8.7%) | 4/6 (66.7%) | 9.33 (2.04 - 117.42) | High |
| **By T2:** |  |  |  |  |
| Volume of practice with internal focus of attention >479.55 hrs by T2 | 5/23 (21.7%) | 5/6 (83.3%) | 7.5 (1.6 - 94.94) | High |
| Volume of practice with external focus of attention >1100.94 hrs | 0/23 (0%) | 2/6 (33.3%) | 9.2 (1.06 - 640.23) | High |
| ***Source of feedback*** |  |  |  |  |
| **By T1:** |  |  |  |  |
| Proportion of intrinsic feedback >21% | 2/23 (8.7%) | 5/6 (83.3%) | 17.5 (3.39 - 293.37) | High |
| Proportion of extrinsic feedback >79% | 21/23 (91.3%) | 1/6 (16.7%) | 0.02 (0 - 0.3) | High |
| **By T2:** |  |  |  |  |
| Proportion of intrinsic feedback >25% | 3/23 (13%) | 5/6 (83.3%) | 12.5 (2.55 - 181.04) | High |
| Proportion of extrinsic feedback >75% | 20/23 (87%) | 1/6 (16.7%) | 0.02 (0.01 - 0.39) | High |
| **Between T1 and T2:** |  |  |  |  |
| ***Prescriptive versus constraints coaching*** |  |  |  |  |
| **By T1:** |  |  |  |  |
| Proportion of practice with prescriptive coaching >49% | 22/23 (95.7%) | 2/6 (33.3%) | 0.02 (0 - 0.36) | High |
| Volume of practice with constraints-based coaching >232.49 hrs by T1 | 0/23 (0%) | 2/6 (33.3%) | 9.2 (1.06 - 640.23) | Moderate |
| **By T2:** |  |  |  |  |
| Proportion of practice with constraints-based coaching >20% | 3/23 (13%) | 4/6 (66.7%) | 6.67 (1.55 - 71.7) | High |
| Proportion of practice with prescriptive coaching >40% | 22/23 (95.7%) | 2/6 (33.3%) | 0.02 (0 - 0.36) | High |
| Volume of practice with constraints-based coaching > 366.24 hrs by T2 | 0/23 (0%) | 2/6 (33.3%) | 9.2 (1.06 - 640.23) | Moderate |
